# Validation of the Disaster Preparedness Evaluation Tool for Nurses—The Korean Version

**DOI:** 10.3390/ijerph18031348

**Published:** 2021-02-02

**Authors:** Suk Jung Han, Jiyoung Chun

**Affiliations:** College of Nursing, Sahmyook University, Seoul 01795, Korea; hansj@syu.ac.kr

**Keywords:** nurses, disasters, prevention, questionnaires, validation, Korea

## Abstract

(1) Background: The purpose of this study was to validate a Korean version of the disaster preparedness evaluation tool (DPET-K) for nurses and to verify its validity and reliability for use in community healthcare centers and hospitals in South Korea; (2) Methods: In total, 497 nurses (248 for exploratory factor analysis and 249 for confirmatory factor analysis) at public health centers, public health sub-centers, public health clinics, and general hospitals in Seoul and Gyeonggi, Chungcheong, and Gangwon Provinces participated in this study. The tool went through translation and back-translation, content validity verification, a pilot survey, and validity and reliability testing; (3) Results: The DPET-K had 28 items with five factors (disaster education and training, disaster knowledge and information, bioterrorism and emergency response, disaster response, and disaster evaluation). The Cronbach’s α values for internal consistency were 0.766–0.953 for the five subscales of the DPET-K. A structural equation model was built through confirmatory factor analysis for goodness of fit (χ^2^/df = 2.193, SRMR = 0.060, RMSEA = 0.069, GFI = 0.831, CFI = 0.927, NFI = 0.875); (4) Conclusions: The DPET-K was confirmed to be a useful tool for assessing the disaster preparedness of nurses in Korea.

## 1. Introduction

Disaster preparedness refers to all action plans and efforts prior to the occurrence of a disaster to establish a disaster response system, which actively involves nurses [1]. Nurses are considered to be experts in disaster management and healthcare, and through their expert knowledge and skills, they provide nursing care that reduces the risk of harm to people’s lives and health caused by disasters [2,3]. Therefore, understanding nurses’ level of disaster preparedness is critical for understanding the status of the disaster management process.

Four main tools have been used to assess the disaster preparedness of nurses in Korea. First, Noh (2010) developed a 44-item tool with reference to the International Council of Nurses (ICN) disaster nursing competencies and the 2003 Wisconsin Health Alert Network Emergency Preparedness Questionnaire (EPIQ) [4]. Second, a tool developed by Kang et al. in 2012 assessed nursing college students’ level of interest in disasters and essential supplies, as well as their awareness regarding the importance of disaster nursing, individual disaster preparedness, and the core competencies of disaster nursing (emergency care, communication, and disaster response system) [5,6]. Third, a tool was developed to measure the disaster preparedness of nurses based on the disaster nursing competencies proposed by the ICN, of which the validity and reliability were verified [7]. Fourth, the disaster preparedness evaluation tool (DPET) was created by Bond and Tichy (2007) to measure nurse practitioners’ knowledge and skills regarding disaster and post-disaster response and management [8].

Bond and Tichy (2007) originally developed the DPET to assess nurse practitioners’ knowledge and skills regarding disaster management [8]. The development of the original DPET was based on the recommended disaster preparedness competencies for nurse practitioners found in the American Association of Colleges of Nursing’s Essentials of Master’s Education (1996) [9]. The DPET has been translated and used in many different countries, including Asian countries such as Japan, Hong Kong, Indonesia, and Taiwan and Middle Eastern nations such as Iran, Jordan, and Saudi Arabia. The studies conducted in Japan [10], Indonesia [11], Iran [12], the Asia-Pacific region [13], and Saudi Arabia [14] focused on finding out the level of nurses’ disaster competency in various stages of the disaster. Meanwhile, the studies conducted in Hong Kong [15], Taiwan [16], and Jordan [17] verified the validity and reliability of the DPET among nurses in their respective countries.

One previous study used the DPET in Korea, and only measured the disaster preparedness capability among public health nurses [18]. Disaster preparedness should be a competency of both public health nurses and nurses at general hospitals. Therefore, this study was conducted to verify the validity and reliability of the DPET among nurses in both public healthcare centers and general hospitals. After adaptations were made to align DPET with Korean culture and language (DPET-K), various methods were used to evaluate it.

## 2. Materials and Methods

### 2.1. Data Collection and Ethical Considerations

Survey data were collected from nurses working at 13 public health centers, three public health sub-centers, 30 public health posts, and two general hospitals with consent from the department heads of each institution. Convenience sampling was used in the process of distributing explanations of the survey, informed consent forms, and survey forms to nurses who volunteered to participate in the survey. The procedure is as follows. After explaining the purpose and method of the research to the institution or department’s head, permission for data collection was obtained. Then, the researcher delivered the questionnaire directly to the head of the institution or department. A person in charge of the institution or department distributed the questionnaires and contacted the researchers after collecting the completed questionnaires. Researchers visited and collected the questionnaires.

### 2.2. Participants

The participants were nurses working at public health centers, public health sub-centers, public health posts, and general hospitals in Seoul and Gyeonggi, Chungcheong, and Gangwon Provinces in Korea and they understood the purpose of this study and provided written consent.

The ideal sample size to determine the validity of a tool is about 5 to 10 times the number of items in the tool as this helps ensure the stability of analyses like factor analysis or item correlation analysis [19]. A total of 550 copies of the survey were distributed among the nurses to examine the construct validity of the initial version of the tool, which consisted of 46 items. Among them, 497 survey responses were used for further analyses, after excluding 39 surveys that were never returned (93% response rate) and 14 responses that did not have sufficient information.

### 2.3. Instruments

The tool consists of items relating to three stages of a disaster: pre-disaster preparedness, mitigation and response, and evaluation. Each item provides responses on a 6-point Likert scale (1 = strongly disagree, 2 = disagree, 3 = somewhat disagree, 4 = somewhat agree, 5 = agree, and 6 = strongly agree, with higher scores representing higher disaster preparedness). The original version Disaster Preparedness Evaluation Tool (DPET) consists of 46 items. The items (25) in the pre-disaster stage probe disaster knowledge, skills, and individual preparedness, while the items (16) in the mitigation and response stage assess knowledge of disaster and patient care. The final six items in the evaluation stage measure readiness for disaster knowledge and management. The Cronbach’s alpha values of each criterion in the original survey were between 0.91 and 0.93.

### 2.4. Validation of the DPET-K Version

This study followed the instrument development guidelines presented by DeVellis [19]. After obtaining permission from Elaine A. Bond to translate and use the tool for this study, the study proceeded in the following order: translation and back-translation, content validity verification, a preliminary survey, the actual survey, item analysis, validity verification, reliability verification, and selection of the final items.

#### 2.4.1. Translation and Back-Translation

The translation was carried out in the following steps: First, the forward-translation of the tool (English to Korean) was completed by an organization specializing in translation. Next, a nursing professor, who is fluent in both Korean and English, drafted the translation. Second, the researcher of this study reviewed the translation to confirm its accuracy as well as the adaptability of the terminology and content in the Korean scenario. Third, a public health nurse and an ER head nurse with experience in disaster education and disaster response provided a consultation to confirm the adaptability of the content. Fourth, a bilingual individual back-translated the content into English. Fifth, a professor (native English speaker) of nursing and public health from the U.S. compared the original and back-translated surveys to confirm that there were no deviations in the meaning of each question. Finally, a professor of the Korean language reviewed the final draft of the translation to fine-tune the meaning and word order.

#### 2.4.2. Content Validity

The DPET-K had the same format as the original tool. A total of seven academic experts and clinical experts were selected to determine the validity of the content. As academic experts, four nursing professors in the fields of community health nursing and adult nursing (specializing in emergency nursing) were selected. As clinical experts, three nurse managers who had worked or were currently working as ER head nurses at tertiary hospitals were selected. Content validity was measured using the content validity index (CVI), using a 4-point scale with the following responses: 1 = not relevant; 2 = revision required: unable to evaluate relevancy or appears not to be relevant without revision; 3 = relevant but requires modest revision; and 4 = highly relevant and concise. In the first round of verification, items were removed if four or more out of the seven experts rated the CVI with 3 or 4 points, reflecting a level of expert agreement less than 80%. This was followed by a second round of content validity verification, in which corrections and/or revisions were made to items that experts indicated were unclear, too long, or unsuitable for the Korean context. In the second round of verification, the group of seven experts reached an agreement of 80% or higher regarding the CVI for all 46 items.

#### 2.4.3. Preliminary Pilot Study

The purpose of a pilot study is to develop and test the acceptability of the study’s instruments. Experts generally recommend 10% of the items as the number of samples to be tested [20]. A pilot study is a small exploratory study for determining the understanding of the items. DPET-K analyzed the validity and reliability, excluding pilot study data.

The DPET-K developed by this study had the same structure, arrangement, and format as Bond and Tichy’s tool [8]. In order to check the format, linguistic expressions, and response time, a pilot survey was conducted among 55 nurses; they were from hospitals and public health centers in the Seoul metropolitan area and provided written consent.

After the pilot survey, there were two requests for the clarification of the definition of a “disaster,” and one concern that the items of the survey instrument were not easy to read. To resolve these issues, the research team added brief descriptions of key terms such as “disaster,” “natural disaster,” “social disaster,” and “disaster preparedness stages” after discussion, so that the definition of a disaster would be clearer to the participants. Through this process, the survey instrument was finalized.

#### 2.4.4. Construct Validity

Exploratory factor analysis (EFA) and confirmatory factor analysis (CFA) were carried out to evaluate construct validity [21,22]. All 497 responses were randomly assigned for use in EFA (248 responses) or CFA (249 responses). The model fit, convergent validity, discriminant validity, and concurrent validity were evaluated.

#### 2.4.5. Reliability

To verify the reliability of the DPET-K, internal consistency was analyzed using Cronbach’s alpha. The Cronbach’s alpha values of the preliminary survey of this study were between 0.94 and 0.96.

### 2.5. Data Analysis Methods

The collected data were analyzed using SPSS (Armonk, NY, USA) and AMOS for Windows (v.22) (Armonk, NY, USA). The general characteristics of the participants were analyzed using descriptive statistics, while construct validity was determined using EFA and CFA. Items were analyzed using item-total correlations, and the Kaiser–Meyer–Olkin (KMO) test and the Bartlett test of sphericity were performed to determine the fit of EFA. When the fit of the model was confirmed, the number of items and factors was decided based on a total explained variance of 60%, eigenvalues of 1.0 or higher in the major component factor analysis, and factor loading of 0.50 or higher using the varimax orthogonal rotation method [19].

Once the number of factors was decided, the goodness of fit of the model was determined using indexes such as χ2/df, root mean square error of approximation (RMSEA), goodness of fit index (GFI), and adjusted goodness of fit index (AGFI) through CFA. Factor loading, significance, average variance extracted (AVE) > 0.50, and construct reliability (CR) > 0.70 were confirmed to verify convergent validity, which reflects whether the items included in the factorial model consistently measured the concepts that were included in the instrument. To verify discriminant validity, the AVE for each factor was checked to determine whether it was larger than the square of the correlation coefficients between the factors [23].

## 3. Results

### 3.1. General Characteristics of the Participants

The participants included 381 public health nurses (76.7%) and 116 hospital nurses (23.3%), and 96.4% (479) of them were women. The average nursing experience of the participants was 11.9 ± 8.8. Among the participants, 64.4% stated that they had participated in disaster education or training. The majority of them (64.2%) said that they received disaster education during on-the-job training. Almost all the participants (94.4%; 469) responded that they would like to receive disaster education. No significant differences were found between the EFA (*N* = 248) and CFA (*N* = 249) groups according to the working place, gender, age, education, marital status, and nursing experience (Table 1).

### 3.2. Item Analysis

The validity of each item was verified using the mean, standard deviation, skewness and kurtosis, and item-total correlation of the 46 items. For skewness, items with absolute values less than 1.96 were selected to ensure normality, and only items with an item-total correlation coefficient of 0.30 or higher were included.

All items of this instrument had skewness values between −1.28 and 1.15, showing a normal distribution, and the communality of all items was also over 0.5. Item-total correlation analysis was conducted to evaluate the correlation between each item and the entire questionnaire. The correlation coefficients for each factor were all higher than 0.30 (range, 0.338–0.776), suggesting a good fit. Internal consistency of the 46 items was confirmed, with a Cronbach’s alpha of 0.96.

### 3.3. Validity Testing

#### 3.3.1. Content Validity

The CVI of all 46 items, as evaluated by the seven experts, showed agreement of 80% or higher.

#### 3.3.2. Construct Validity

##### Exploratory Factor Analysis

In the EFA group (*N* = 249), the goodness of fit of the model was confirmed prior to factor analysis by the KMO test and the Bartlett sphericity test. The KMO value was calculated to be 0.948, which indicated a suitable fit for factor analysis, and the chi-square value of the Bartlett sphericity test was 9601.826 (*p* < 0.001). Factor analysis was performed after confirming the goodness of fit of the model.

Eight factors were extracted as a result of the first factor analysis, with a total explained variance of 70.41% and then, seven items with a factor loading were lower than 0.5 (items 6, 14, and 29) or a factor loading of 0.4 or higher for two or more factors (items 27, 35, 38, and 39). Those items were not statistically significant. Moreover, item 6 (I am aware of classes about disaster preparedness and management that are offered, for example, at my workplace, the university, or community) was considered to be included in item 4 (I participate in one of the following educational activities on regular basis: continuing education classes, seminars, or conferences dealing with disaster preparedness) in Korea and deleted. The nurses of Korea currently are considered to be healthcare providers limited to the roles of a nurse and a team member [24,25]. So, item 14 (In case of a disaster situation, I think that there is sufficient support from local officials on the county or state level), and 38 (I am familiar with the organizational logistics and roles among local, state, and federal agencies in disaster response situations) were deleted. Nurses at hospitals and even nurses at public health centers in Korea do not perform the roles, as nurses cannot participate in government policy-making and implementation or play the role of a community leader. For the same reason, item 39 was deleted. Item 27 (I can manage the common symptoms and reactions of disaster survivors that are of affective, behavioral, cognitive, and physical nature) included PTSD. Disasters in Korea are managed by the government control system [24]. The general public and media all controlled by government. Considering this, item 29 (I am able to describe my role in the response phase of a disaster in the context of my workplace, the general public, media, and personal contacts) was discarded. In the scenario of a bioterrorism/biological attack, health history and assessment are conducted by a special control system [24]. Therefore, general nurses are not assigned the task and item 35 (In case of a bioterrorism/biological attack, I know how to perform focused health history and assessment, specific to the bioagents that are used) was discarded. As stated above, various items were discarded considering the situation in Korea and finally, 39 items remained.

Six factors were extracted in the second round of factor analysis, with a total explained variance of 66.698%. Then, three items were with a factor loading lower than 0.5 (item 26), a factor loading of 0.4 or higher for two or more factors (item 16), or a factor loading of 0.5 or lower for communality (item 15). Korea has a disaster medical response system which is in a centralized top-down format, and thus, nurses only provide the nursing care [25]. Item 15 (I participate/have participated in creating new guidelines, emergency plans, or lobbying for improvements on the local or national level), and 16 (I would be considered a key leadership figure in my community in a disaster situation) were discarded for this reason. The Korean nurses follow the procedures rather than identifying indicators of mass exposure according to the disaster medical response system [25]; therefore, item 26 (I can identify possible indicators of mass exposure evidenced by a clustering of patients with similar symptoms) was deleted. Items 5 (I read journal articles related to disaster preparedness) and 7 (I would be interested in educational classes on disaster preparedness that relate specifically to my community situation), which were grouped together as a single factor, were also removed because those items contained the basic meaning of disaster preparedness and could be included in item 4.

After this process, the final questionnaire consisted of 34 items and five factors. The percentage of explained variance for each factor was 19.2% for recovery, 17.6% for bio-terrorism and emergency response, 14.0% for disaster knowledge and information, 8.3% for disaster response, and 7.9% for disaster education and training, with a total explained variance of 66.949% (Table 2).

##### Confirmatory Factor Analysis

Model fit was confirmed by structural equation modeling of the five factors determined from EFA and the items in each factor. The indexes (χ^2^/df = 4.401, RESEA = 0.083, GFI = 0.805, and CFI = 0.886) fell short of the standard. Six items (17, 23, 24, 25, 30, 31) that had high correlations with multiple factors or were similarly correlated with two factors were informed, with reference to the modification index results. Item 17 (I am aware of what the potential vulnerabilities in my community are, e.g., earthquake, floods, terror, etc.) was included in item 18 (I know the limits of my knowledge, skills, and authority as an RN to act in disaster situations, and I would know when I exceed them). Item 23 (I am familiar with accepted triage principles used in disaster situations) was considered to be included in item 36 (I would feel confident working as a triage nurse practitioner, and setting up temporary clinics in disaster situations) and deleted. Since the family’s disaster plan was judged to be less related to the nurse‘s disaster readiness, item 24 (I have personal/family emergency plans in place for disaster situations), and item 25 (I have an agreement with loved ones and family members on how to execute our personal/family emergency plans) were deleted. Diagnosis and treatment in bio-terrorism situations of Korea are performed by the disaster medical assistance team according to the disaster medical response system [25]. So, item 30 (I am familiar with the main Groups (A, B, C) of biological weapons (anthrax, plague, botulism, smallpox, etc.), their signs and symptoms, and effective treatments), and item 31 (I feel confident discerning deviations in health assessments indicating potential exposure to biological agents.) including ‘bio-terrorism and response’ were deleted.

The total number of items was reduced to 28 and the absolute fit indexes (χ^2^/df = 2.193, SRMR = 0.060, RMSEA = 0.069, GFI = 0.831, CFI = 0.927, and NFI = 0.875) aligned with the recommended values of GFI and CFI close to 1.00, SRMR ≤ 0.05, and RMSEA from 0.8 to 0.10 [23] (Table 3).

##### Convergent Validity

Convergent validity was examined to determine the shared variance of any latent variables. The composite reliability (CR) of the DPET-K items ranged from 0.659 to 0.937; and the AVE ranged from 0.413 to 0.710. (Table 3).

##### Discriminant Validity

Discriminant validity is considered to be present if the AVEs are greater than the squared correlation coefficient. All AVEs were observed to be greater than the square of the correlation coefficient between each factor. Thus, discriminant validity was supported; that is, each factor was found to be different from all other factors (Table 4).

##### Concurrent Validity

The disaster nursing tool developed by Noh (2010) was chosen for correlational analysis [4]. For criterion validity, items are considered to be correlated if the correlation coefficient is between 0.40 and 0.60, and closely correlated if it is between 0.60 and 0.80. The correlations between the items of this tool and Noh’s (2010) tool ranged from r = 0.481 (*p* < 0.001) to 0.740 (*p* < 0.001) (Table 5).

### 3.4. Reliability and Mean Scores for Each Factor

The internal consistency of the items of the five factors and item score distributions were calculated. Cronbach’s alpha for all 28 items was 0.954. For each subdomain, the values were 0.766 for the four items on disaster education and training, 0.892 for the seven items on disaster knowledge and information, 0.926 for the four items on bioterrorism and emergency response, 0.886 for the five items on disaster response, and 0.953 for the eight items on disaster evaluation. The mean scores were calculated for each of the 28 items and they ranged from 2.92 to 4.52 (Table 6).

## 4. Discussion

This study examined the properties of DPET-K and various methods were used to assess its content, construct validity, and reliability. The tool consists of five factors and 28 items, and its reliability and validity were confirmed through a survey of Korean community health nurses and hospital nurse practitioners. The findings suggest that DPET-K is structurally valid with five distinct yet convergent subscales and possesses internal reliability. This study is meaningful in the sense that it streamlined the tool by discarding items not suitable to the Korean context and organizing items in a statistically significant way, improving the utility of the tool.

It is also notable that this study was the first to conduct factor analyses of a version of the widely used DPET tool specifically targeting Korean nurses. In particular, the translation and back-translation were based on consultations with nurses who had experience in disaster situations, which led to the development of a final version that can be understood by all nurses working in public healthcare centers and hospitals. The content validity of this tool for both theoretical and clinical aspects of disaster preparedness was verified by four professors with expertise in disaster nursing and three clinical head nurses. The questionnaire was also revised after a pilot survey of nurses to ensure that it could be easily understood both theoretically and clinically, and it was established that this tool can be utilized in a broad range of public healthcare and hospital settings.

The DPET-K is suitable for measuring the disaster preparedness of nurses in Korea and is adapted to their culture and society; however, we also inferred that several additions/improvements were needed in the nurses’ role in the future. First, the role of nurses should extend to coordinators, organizers, and leaders. Disaster management in Korea focuses on the disaster response system of the government [24,25]. For this reason, various roles of nurses in disasters covered in the original DPET were excluded in DPET-K. This foregrounded the absence of nurses in policy positions, which were included in the role of nurses in disaster by the international council of nurses [26]. Second, the role of a nurse needs to include daily life and community. On this regard, Korean society is making a distinction between a nurse’s role and that of the individual/family. For the same reason, disaster preparedness of nurses in Korean nurses was based on the workplace. However, it would will be difficult to perform active disaster nursing if individuals and families are not prepared. Therefore, disaster preparation for individuals/family needs to be introduced. Third, it is necessary to provide general nurses with more information and skill about the symptoms, assessment, and treatment related to the disaster. Since Korea has a separate disaster medical assistant team (DMAT) dedicated to disasters, it was difficult to cover all aspects of disaster nursing for general nurses working in public health centers and hospitals through DPET-K. But in a pandemic situation such as COVID-19, it is imperative that all nurses participate in disaster nursing.

In this way, the DPET-K has been reorganized in the Korean situation. However, there are many aspects to be considered as in the original DPET by referring to the necessary elements of disaster nursing. Nevertheless, DPET-K has become closer in terms of cultural accessibility for nurses working in various field in Korea. Therefore, DPET-K is considered to be a useful tool to measure the disaster preparedness of Korean nurses of public healthcare centers and hospitals.

In a disaster situation, nurses’ leadership and empowerment strengthen interprofessional collaboration and help solve problems [27]. Education to enhance nurses’ disaster preparedness, such as an education curriculum reflecting the ICN framework, is essential [28]. In particular, this education encompasses practical as well as theoretical knowledge [29,30]. Therefore, to increase Korean nurses’ disaster preparedness, it is necessary to develop an applicable curriculum that grants nurses leadership and authority during a disaster. Through this, it is thought that nurses’ core competence can be improved to increase disaster preparedness [31].

### Limitation

This study adapted and translated Bond and Tichy’s DPET and verified the resulting DPET-K using EFA and CFA. Some items were removed because they did not reflect Korea’s circumstances and did not satisfy the criteria for statistical power. DPET-K was identified through statistical analysis, and the final decision was made based on Korean cultural suitability. In the EFA, the disaster data for items 5, 6, and 7 and patient symptom management for item 27 were excluded for statistical power in that they could be included in other items. Items 14, 15, and 16 were excluded because the general nurse’s role in Korea is limited to team members. Items 26, 29, 35, 38, and 39 were excluded because they could not be generalized to all Korean nurses because it is the Korean government’s role. In the CFA, items 17, 23, 30, and 31 were excluded in the aspect that they could be included in other items. Items 24 and 25, related to the family’s disaster readiness, were also excluded because they were considered task-oriented. Therefore, adding and verifying other items reflecting Korea-specific factors to the original DPET could be another way to develop the tool.

DPET-K examines only the perceptions of nurses participating in the study, as it is challenging to measure disaster readiness in real situations accurately. Therefore, attention should be paid to the purpose and interpretation of DPET-K. Moreover, since this study was only for nurses, it is necessary to expand and verify the DPET-K to include various occupations like disaster paramedics.

## 5. Conclusions

This study adapted the DPET to the Korean context, determined its validity and reliability, and streamlined its content. The original DPET was condensed into five factors and 28 items. The DPET-K consists of a three-phase pre-disaster stage, disaster stage, and post-disaster stage. The pre-disaster stage (prevention) has 15 items composed of disaster education, disaster knowledge, and bioterrorism; the disaster stage (mitigation) has five disaster response items; and the post-disaster stage (recovery) has nine items of disaster evaluation. The DPET-K is expected to help understand disaster preparedness at each stage and analyze and interpret each of the five factors.

## Figures and Tables

**Table 1:** General characteristics of participants (*N* = 497).

Variables	Categories	Total		EFA (*N* = 248)		CFA (*N* = 249)		χ^2^ (p)
		*N*	(%)	*N*	(%)	*N*	(%)	*t*-test (*p*)
Working place	Public health	381	(76.7)	190	(76.3)	191	(77.0)	0.111 (0.797)
	Hospital	116	(23.3)	59	(23.7)	57	(23.0)	
Gender	Women	479	(96.4)	236	(94.8)	243	(98.0)	0.190 (0.772)
	Men	18	(3.6)	13	(5.2)	5	(2.0)	
Age	Mean ± SD	38.6 ± 9.9		38.1 ± 9.8		39.1 ± 10.1		0.037 (0.971)
	≤29	118	(23.7)	65	(26.1)	53	(21.4)	
	30~39	168	(33.8)	82	(32.9)	86	(34.7)	
	40~49	120	(24.2)	60	(24.1)	60	(24.2)	
	≥50	91	(18.3)	42	(16.9)	49	(19.8)	
Education	3-year college	163	(32.8)	78	(31.3)	85	(34.3)	2.539 (0.292)
	4-year university	288	(57.9)	148	(59.4)	140	(56.5)	
	Master’s degree	40	(8.1)	21	(8.4)	19	(7.7)	
	Doctoral degree	6	(1.2)	2	(0.8)	4	(1.6)	
Marital status	Not married	171	(34.4)	87	(34.9)	84	(33.9)	2.473 ^†^ (0.546)
	Married	320	(64.4)	160	(64.3)	160	(64.5)	
	Divorced	2	(0.4)	1	(0.4)	1	(0.4)	
	Bereaved	4	(0.8)	1	(0.4)	3	(1.2)	
Nursing experience (unit: year)	Mean ± SD	11.9 ± 8.8		11.1 ± 8.6		12.7 ± 9.0		0.879 (0.380)
	≤5	127	(25.6)	73	(29.3)	54	(21.8)	
	6~10	144	(29.0)	71	(28.5)	73	(29.4)	
	11~15	83	(16.7)	43	(17.3)	40	(16.1)	
	16~20	57	(11.5)	25	(10.0)	32	(12.9)	
	21~25	29	(5.8)	13	(5.2)	16	(6.5)	
	26~30	34	(6.8)	15	(6.0)	19	(7.7)	
	≥31	22	(4.4)	9	(3.6)	13	(5.2)	
Participation disaster education and drills	Yes	320	(64.4)	149	(59.8)	171	(68.9)	1.160 (0.700)
	No	177	(35.6)	100	(40.2)	77	(31.0)	
When respondent was educated about disaster preparedness ^‡^	Undergraduate nursing program	135	(27.2)	75	(30.1)	60	(24.2)	-
	Graduate nursing program	24	(4.8)	9	(3.6)	15	(6.0)	
	Drills in the workplace	319	(64.2)	149	(59.8)	170	(68.5)	
	Continuing education courses	92	(18.5)	39	(15.7)	53	(21.4)	
Desired disaster education ^‡^	Nurse’s role in a disaster situation	323	(65.0)	170	(68.3)	153	(61.7)	-
	Biological agents and ways to identify their signs and symptoms	280	(56.3)	147	(59.0)	133	(53.6)	
	How to respond in community settings in case of disaster	263	(52.9)	145	(58.2)	118	(47.6)	
	Resources such as agencies for referral, the chain of command, and community shelters	248	(49.9)	130	(52.2)	118	(47.6)	
	Recovery state: PTSD ^§^, acute stress disorder, crisis intervention	241	(48.5)	130	(52.2)	111	(44.8)	
	Biological agents and their differential diagnosis and treatments	211	(42.5)	119	(47.8)	92	(37.1)	
	Potential vulnerabilities existing in the country in case of a disaster	162	(32.6)	94	(37.8)	68	(27.4)	

^†^ Fisher’s exact test, ^‡^ Multiple responses, ^§^ PTSD: Post-traumatic stress disorder.

**Table 2:** Explanatory factor analysis (*N* = 248).

Items	Factor				
	1	2	3	4	5
46	0.865	0.206	0.190	0.089	0.100
45	0.863	0.222	0.187	0.072	0.102
42	0.840	0.128	0.201	0.188	0.064
41	0.819	0.166	0.205	0.271	0.054
40	0.708	0.200	0.267	0.384	0.090
44	0.703	0.325	0.078	0.189	0.182
43	0.692	0.233	0.209	0.363	0.209
28	0.623	0.240	0.361	0.247	0.076
20	0.194	0.825	0.246	0.108	0.141
21	0.218	0.811	0.260	0.167	0.136
19	0.109	0.728	0.298	0.225	0.160
22	0.230	0.637	0.358	0.160	0.284
30	0.369	0.609	0.200	0.190	0.019
24	0.310	0.604	0.332	0.115	0.109
31	0.370	0.540	0.192	0.334	0.003
25	0.333	0.532	0.326	0.065	0.044
23	0.178	0.455	0.170	0.242	0.376
9	0.196	0.210	0.731	0.087	0.117
8	0.211	0.268	0.715	−0.039	0.162
12	0.187	0.299	0.707	0.164	0.137
10	0.230	0.241	0.652	0.070	0.302
11	0.216	0.207	0.650	0.175	0.176
13	0.112	0.183	0.608	0.304	0.220
17	0.142	0.187	0.546	0.297	0.202
18	0.258	0.305	0.534	0.359	0.198
33	0.335	0.195	0.230	0.754	0.113
32	0.275	0.112	0.248	0.752	0.038
36	0.325	0.299	0.167	0.583	0.193
34	0.330	0.419	0.130	0.542	0.039
37	0.376	0.370	0.088	0.527	0.159
2	0.159	0.222	0.027	−0.018	0.700
3	0.049	0.140	0.345	0.223	0.700
1	0.001	−0.050	0.268	0.161	0.681
4	0.151	0.158	0.273	−0.037	0.659
Eigenvalue	6.520	5.974	4.768	2.806	2.696
Variance (%)	19.176	17.570	14.023	8.252	7.928
Cumulative variance (%)	19.176	36.746	50.769	59.021	66.949

Note: Significant factor loadings are in background.

**Table 3:** Convergent validity and model fitness according in the confirmatory factor analysis (*N* = 249).

Phase	Factor	Item	Unstandardized Estimates	SE ^†^	CR ^‡^	Standardized Estimate	AVE ^§^	C.R ^‖^
Prevention (pre-disaster stage)	Disaster education and training (4 items)	Factor5→P4	1			0.663	0.413	0.659
		Factor5→P3	1.112	0.109	10.156	0.838		
		Factor5→P2	0.898	0.12	7.486	0.551		
		Factor5→P1	0.857	0.094	9.131	0.698		
	Disaster knowledge and information (7 items)	Factor3→P18	1			0.652	0.710	0.896
		Factor3→P13	1.241	0.128	9.677	0.711		
		Factor3→P11	1.215	0.12	10.098	0.749		
		Factor3→P10	1.22	0.12	10.135	0.752		
		Factor3→P12	1.312	0.124	10.555	0.792		
		Factor3→P8	1.123	0.119	9.397	0.687		
		Factor3→P9	1.133	0.119	9.504	0.697		
	Bio-terrorism and emergency response (4 items)	Factor2→P22	1			0.766	0.591	0.847
		Factor2→P19	1.252	0.083	15.019	0.869		
		Factor2→P21	1.307	0.08	16.396	0.933		
		Factor2→P20	1.283	0.079	16.315	0.929		
Mitigation (disaster stage)	Disaster response (5 items)	Factor4→P37	1			0.819	0.542	0.857
		Factor4→P34	0.971	0.066	14.688	0.818		
		Factor4→P36	1.017	0.07	14.589	0.814		
		Factor4→P32	0.686	0.067	10.3	0.624		
		Factor4→P33	0.854	0.066	12.877	0.743		
Recovery (post-disaster stage)	Disaster evaluation (8 items)	Factor1→P28	1			0.777	0.690	0.937
		Factor1→P43	1.086	0.072	15.135	0.854		
		Factor1→P40	1.239	0.077	16.059	0.893		
		Factor1→P44	0.903	0.073	12.365	0.728		
		Factor1→P41	1.207	0.073	16.431	0.908		
		Factor1→P42	1.164	0.075	15.549	0.872		
		Factor1→P46	1.122	0.073	15.365	0.864		
		Factor1→P45	1.096	0.073	14.923	0.845		
Total 28 items	χ^2^/df = 2.193, SRMR ^¶^ = 0.060, RMSEA ^††^ = 0.069, GFI ^§§^ = 0.831, CFI ^‖^ = 0.927, NFI ^¶¶^ = 0.875							

^†^ SE: standard error; ^‡^ CR: critical ratio; ^§^ AVE: average variance extracted estimate; ^‖^ CR: construct reliability; ^¶^ SRMR: standardized root mean-square residual; ^††^ RMSEA: root mean square error of approximation; ^§§^ GFI: goodness of fit index; ^‖^ CFI: comparative fit index; ^¶¶^ NFI: normed fit index.

**Table 4:** Discriminant validity in the confirmatory factor analysis (*N* = 248).

Variable	Factor 1	Factor 2	Factor 3	Factor 4	Factor 5
	r(p)	r(p)	r(p)	r(p)	r(p)
Factor 1	0.690 *				
Factor 2	0.385 (<0.001)	0.710 *			
Factor 3	0.434 (<0.001)	0.412 (<0.001)	0.591 *		
Factor 4	0.694 (<0.001)	0.457 (<0.001)	0.603 (<0.001)	0.542 *	
Factor 5	0.333 (<0.001)	0.450 (<0.001)	0.040 (<0.001)	0.442 (<0.001)	0.413 *
Mean ± SD	3.125 ± 1.008	3.473 ± 0.869	3.089 ± 1.138	3.424 ± 0.952	3.822 ± 1.035

* Values along the diagonal line indicate the average variance extracted.

**Table 5:** Concurrent validity between the Korean disaster preparedness evaluation tool and Noh’s scale (*N* = 248).

DPET-K	Factor 1 r (*p*)	Factor 2 r (*p*)	Factor 3 r (*p*)	Factor 4 r (*p*)	Factor 5 r (*p*)
Factor 1	1				
Factor 2	0.589 (<0.001)	1			
Factor 3	0.724 (<0.001)	0.610 (<0.001)	1		
Factor 4	0.537 (<0.001)	0.631 (<0.001)	0.621 (<0.001)	1	
Factor 5	0.349 (<0.001)	0.576 (<0.001)	0.407 (<0.001)	0.435 (<0.001)	
Noh’s scale	0.729 (<0.001)	0.630 (<0.001)	0.740 (<0.0001)	0.654 (<0.001)	0.481 (<0.001)

**Table 6:** Final item analysis by factor (*N* = 248).

Phase	Factor	No	Items	Mean	SD	Adjusted Item-Total Correlation	Cronbach’s α If Item Deleted	Cronbach’s α
**Prevention** (pre-disaster stage)	Disaster education and training (4 items)	4	I participate in one of the following educational activities on regular basis: continuing education classes, seminars, or conferences dealing with disaster preparedness.	3.28	1.42	0.541	0.724	0.766
		3	I know who to contact (chain of command) in disaster situations in my community.	4.06	1.25	0.697	0.646	
		2	I have participated in emergency plan drafting and emergency planning for disaster situations in my community.	3.38	1.55	0.485	0.763	
		1	I participate in disaster drills or exercises at my workplace (clinic, hospital, etc.) on a regular basis.	4.52	1.22	0.576	0.708	
	Disaster knowledge and information (7 items)	18	I know the limits of my knowledge, skills, and authority as an RN to act in disaster situations, and I would know when I exceed them.	3.59	1.10	0.632	0.883	0.892
		13	I have a list of contacts in the medical or health community in which I practice. I know referral contacts in case of a disaster situation (health department, e.g.,).	3.75	1.21	0.634	0.884	
		11	Finding relevant information about disaster preparedness related to my community needs is an obstacle to my level of preparedness	3.36	1.12	0.710	0.874	
		10	I consider myself prepared for the management of disasters.	3.24	1.12	0.710	0.874	
		12	I know where to find relevant research or information related to disaster preparedness and management to fill in gaps in my knowledge.	3.49	1.14	0.748	0.869	
		8	I find that the research literature on disaster preparedness and management is easily accessible.	3.27	1.13	0.690	0.877	
		9	I find that the research literature on disaster preparedness is understandable.	3.58	1.12	0.708	0.874	
	Bio-terrorism and emergency response (4 items)	22	I am familiar with the local emergency response system for disasters.	3.16	1.19	0.719	0.937	0.926
		19	In case of a bioterrorism/biological attack, I know how to use personal protective equipment.	3.24	1.31	0.821	0.906	
		21	In a case of bioterrorism/biological attack I know how to perform isolation procedures so that I minimize the risks of community exposure.	3.03	1.27	0.887	0.883	
		20	In case of a bioterrorism/biological attack I know how to execute decontamination procedures.	2.92	1.26	0.888	0.883	
**Mitigation** (disaster stage)	Disaster response (5 items)	37	I feel reasonably confident can treat patients independently without supervision of a physician in a disaster situation.	2.93	1.19	0.730	0.861	0.886
		34	As an RN, I would feel reasonably confident in my abilities to be a member of a decontamination team.	3.12	1.15	0.752	0.855	
		36	I would feel confident working as a triage nurse practitioner, and setting up temporary clinics in disaster situations.	3.31	1.21	0.719	0.863	
		32	As an RN, I would feel confident in my abilities as a direct care provider and first responder in disaster situations.	4.04	1.07	0.661	0.876	
		33	As an RN, I would feel confident as a manager or coordinator of a shelter.	3.73	1.12	0.766	0.852	
**Recovery** (post-disaster stage)	Disaster evaluation (8 items)	28	I am familiar with psychological interventions, behavioral therapy, cognitive strategies, support groups and incident debriefing for patients who experience emotional or physical trauma.	3.38	1.14	0.748	0.952	0.953
		43	I am familiar with what the scope of my role as a nurse practitioner in a post-disaster situation would be.	3.35	1.13	0.827	0.947	
		40	I would feel confident providing patient education on stress and abnormal functioning related to trauma.	3.31	1.23	0.847	0.946	
		44	I participate in peer evaluation of skills on disaster preparedness and response.	2.89	1.10	0.708	0.954	
		41	I would feel confident providing education on coping skills and training for patients who experience traumatic situations so they are able to manage themselves.	3.14	1.18	0.875	0.944	
		42	I am able to discern the signs and symptoms of acute stress disorder and post traumatic stress syndrome (PTSD).	3.07	1.19	0.851	0.945	
		46	I feel confident managing (treating, evaluating) emotional outcomes for acute stress disorder or PTSD following disaster or trauma in a multi-disciplinary way such as referrals, and follow-ups and I know what to expect in ensuing months.	2.89	1.16	0.878	0.944	
		45	I am familiar with how to perform focused health assessment for PTSD.	2.97	1.15	0.865	0.944	
Total	28 items							0.954
